# A new method to compute K-mer frequencies and its application to annotate large repetitive plant genomes

**DOI:** 10.1186/1471-2164-9-517

**Published:** 2008-10-31

**Authors:** Stefan Kurtz, Apurva Narechania, Joshua C Stein, Doreen Ware

**Affiliations:** 1 Center for Bioinformatics, University of Hamburg, Bundesstraße 43, 20146 Hamburg, Germany; 2 Cold Spring Harbor Lab, 1 Bungtown Rd, Williams #5, Cold Spring Harbor, NY 11724, USA; 3 Sackler Institute for Comparative Genomics, American Museum of Natural History, New York, NY, USA

## Abstract

**Background:**

The challenges of accurate gene prediction and enumeration are further aggravated in large genomes that contain highly repetitive transposable elements (TEs). Yet TEs play a substantial role in genome evolution and are themselves an important subject of study. Repeat annotation, based on counting occurrences of *k*-mers, has been previously used to distinguish TEs from low-copy genic regions; but currently available software solutions are impractical due to high memory requirements or specialization for specific user-tasks.

**Results:**

Here we introduce the Tallymer software, a flexible and memory-efficient collection of programs for *k*-mer counting and indexing of large sequence sets. Unlike previous methods, Tallymer is based on enhanced suffix arrays. This gives a much larger flexibility concerning the choice of the *k*-mer size. Tallymer can process large data sizes of several billion bases. We used it in a variety of applications to study the genomes of maize and other plant species. In particular, Tallymer was used to index a set of whole genome shotgun sequences from maize (B73) (total size 10^9 ^bp.). We analyzed *k*-mer frequencies for a wide range of *k*. At this low genome coverage (≈ 0.45×) highly repetitive 20-mers constituted 44% of the genome but represented only 1% of all possible *k*-mers. Similar low-complexity was seen in the repeat fractions of sorghum and rice. When applying our method to other maize data sets, High-*C*_0_*t *derived sequences showed the greatest enrichment for low-copy sequences. Among annotated TEs, the most highly repetitive were of the Ty3/gypsy class of retrotransposons, followed by the Ty1/copia class, and DNA transposons. Among expressed sequence tags (EST), a notable fraction contained high-copy *k*-mers, suggesting that transposons are still active in maize. Retrotransposons in Mo17 and McC cultivars were readily detected using the B73 20-mer frequency index, indicating their conservation despite extensive rearrangement across cultivars. Among one hundred annotated bacterial artificial chromosomes (BACs), *k*-mer frequency could be used to detect transposon-encoded genes with 92% sensitivity, compared to 96% using alignment-based repeat masking, while both methods showed 92% specificity.

**Conclusion:**

The Tallymer software was effective in a variety of applications to aid genome annotation in maize, despite limitations imposed by the relatively low coverage of sequence available. For more information on the software, see .

## Background

Repetitive elements abound in the genomes of higher organisms. Tandem repeats, simple sequence repeats, long terminal repeats (LTRs), segmental duplications, and transposable elements (TEs) are among those types commonly found in eukaryotic species. The biological role of these entities in genome evolution has been documented [1,2], but from a computational standpoint, the frequency with which repetitive elements occur may confound gene finding and the alignment of homologous sequences.

For anticipated plant genome projects and those currently underway, effective and rapid annotation of their many repeats has acquired a new urgency. For example, the maize genome is estimated to be 60–70% repetitive, mainly in the form of retrotransposons that proliferated in the last 2 to 6 million years [3-5]. Extensive repeats in maize has required a BAC-by-BAC sequencing approach with finishing primarily focused on the unique space. In this context, computational methods designed to annotate unique sequences have become indispensable.

Repeat identification strategies fall under two broad categories: de novo detection and similarity-based detection. RepeatMasker [6], one of the most widely used computational tools in repeat analysis, employs the latter method and is therefore reliant on precompiled repeat databases specific to the genome in question. RepeatMasker is an essential annotation tool for organisms whose repeat content has been well characterized. However, for many novel genomes a specific repeat library is either nonexistent or insufficient.

De novo methods address many of these concerns. RECON [7] currently the dominant tool for de novo repeat detection, builds a set of repeat families through pairwise similarity, clustering and boundary calling. RECON and other de novo detection programs like REPuter [8], Vmatch [9], RepeatFinder [10], RepeatGluer [11], and PILER [12], are designed to generate a repeat library rather than to use one. But to define repeat families, these self-alignment approaches are best suited to genomes that have been assembled into sizable scaffolds. If sequence is not available at that depth or with that contiguity, de novo methods may prove inaccurate especially with respect to repeat boundaries.

Other more recent programs like ReAS [13] and RepeatScout [14] attempt to build repeat families around frequent *k*-mers. *K*-mer frequency based methods were originally used in whole genome shotgun assemblers [15-17] that omit reads containing frequent *k*-mers for the purposes of assembly. Programs like ReAS operate in an inverse fashion, using only high copy reads to seed a sophisticated repeat finding strategy. But these methods, like genome assemblers, require significant sequence depth to assure adequate coverage over repeat families. Though we propose a similar strategy, our goal is not to identify repeat families. We have found that even if available sequences cover only a fraction of the genome, *k*-mer frequencies alone capture rich statistical information on the repeat profiles of plant genomes.

Like other *k*-mer frequency approaches [18-20] our method does not require a precompiled repeat library. Rather than simple hashing methods (which only work for small values of *k*), we employ enhanced suffix arrays [21] to compute occurrence counts and construct an index (called *k*-mer frequency index) from which we can efficiently retrieve the frequency of each stored *k*-mer. This strategy allows us to process very large datasets for a wide variety of values of *k*, and to assign frequency annotations with unprecedented speed. We developed this method in the context of the Maize Genome Sequencing Project where rapid genome-scale frequency annotation is integral to genome finishing. The project employs a traditional BAC-by-BAC sequencing strategy, but directed sequence finishing will proceed only in those regions designated as unique with respect to a 0.45 × WGS data set generated by the Joint Genome Institute (see Section "Maize WGS data set"). During development of this novel finishing strategy we expanded *k*-mer frequencies into a powerful comparative genomics tool, highlighting the differences in complexity and overall repetitiveness in several grass genomes.

## Methods

### Sequence data sets

This study used publicly available datasets, including expressed sequence tags (ESTs), gene-enriched genome fractions, representative whole genome sequences, and repeat libraries, as summarized in Table 1. In addition, we sequenced and assembled four BAC clones from maize (B73) chromosome 8, which map to fingerprint contig ctg362 of the AGI agarose FPC map [22,23]. Sublibrary construction, sequencing on ABI 3700 machines, and assembly were essentially as described in [24]. Clone names and their GenBank accession numbers are as follows: ZMMBBb0483G05 [Genbank: AC157776], ZMMBBb0284N04 [Genbank: AC157977], ZMMBBb0614J24 [Genbank: AC157487], and ZMMBBb0448F23 [Genbank: AC160211]. A polymerase chain reaction was used on template ZMMBBb0382K21 to give a unique 1864 bp sequence [Genbank: AC163004] that served to fill a physical gap between ZMMBBb0483G05 and ZMMBBb0284N04. Overlap between clone sequences allowed assembly of a complete supercontig of 453,421 bp.

**Table 1:** The different sequence sets used in the validation experiment.

class	fraction	abbrev.	size	source
WGS	Pilot Bacterial Artificial Chromosomes	BAC	14.8	[35]
WGS	BAC End Sequences	BES	8.0	AGI [50]

Repeat	Transposable Elements	RepI	8.0	MIPS [28]
Repeat	DNA transposons	RepII	0.1	MIPS [28]

GE	Expressed Sequence Clusters	EST	56.3	PlantGDB [51]
GE	AZM4 High-*C*_0_*t*	AZM4 HC	188.8	TIGR [52]
GE	AZM4 Methyl-filtered	AZM4 MF	156.8	TIGR [52]

GA	TIGR4 assembly of rice (*Oryza sativa L. ssp. japonica*)	Osj:TIGR4	420.0	TIGR [37]
GA	TAIR7 assembly of *Arabidopsis thaliana*	At:TAIR7	115.4	TAIR [42]
GA	JGI1.1 assembly of poplar (*Populus trichocarpa*)	Pt:JGI1.1	410.0	[43]
GA	Genoscope1 assembly of grapevine (*Vitis vinifera*)	Vc:GEN1	487.0	[44]

WGS stands for survey of 'Whole Genome Sequences'. GE stands for 'gene enrichment'. GA stands for 'genome assemblies'. The sequence sizes are given in million base pairs.

### Maize WGS data set

Whole-genome shotgun reads for Maize (*Zea mays L. ssp. mays*), generated by the Joint Genome Institute were downloaded from the NCBI Trace Archive on February 13, 2006 and clipped to remove vector sequences using cross match [25] and the NCBI UniVec database [26]. The resulting dataset, called maize 0.45 × WGS, included 1,124,441 reads and 1,088,525,270 nucleotides.

### Gene annotation

*Ab initio *gene prediction was conducted using FGE-NESH (monocot matrix) on non-masked BAC sequences. Resulting predictions were subjected to BLASTP against the NCBI non-redundant Gen-Pept database with the low-complexity filter turned on, and presumptive genes were identified as having an E-value ≤ 1*e*^-5^. Transposable elements were screened out by matching their hits against a manually curated list of 2852 transposable element genes.

### Annotation of transposable elements

Transposable elements were identified using Repeat-Masker [6] and MIPS Repeat Element Database (mips-REdat) and Catalog (mips-REcat) [27,28]. This database provides a hierarchical classification of plant transposable elements and other repeat types. Before use, the database was screened for non-TE related sequences and the following identifiers were removed: 'bsr1' (containing cytochrome P450 and hydrolase sequences), 'k_1' (containing proton-ATPase sequence), and 'magellan_cone' (containing myb transcription factor sequence).

Receiver operating characteristic (ROC) curves were compared by the method of [29], as implemented in the MedCalc^® ^statistical software package.

### Basic notions for sequence processing

We consider sequences over the DNA alphabet {A, C, G, T, N}, where N denotes an undetermined base (usually represented by one of the IUPAC characters *S*, *Y*, *W*, *R*, *K*, *V*, *B*, *D*, *H*, *M*, *N*). For fixed length *k *> 0, a *k*-mer is a sequence of length *k*, only containing the characters A, C, G, and T. Let *v *be some sequence. mer_*k*_(*v*) denotes the set of all different *k*-mers in *v*. For any set *M*, |*M*| denotes the number of elements in *M*. So |mer_*k*_(*v*)| is the number of different *k*-mers in *v*. For any sequence *w *of length *k*, let *occ*(*w, v*) be the number of positions in *v *where *w *occurs. For a set *S *of sequences we define mer_*k*_(*S*) = ∪_*v*∈*S *_mer_*k*_(*v*) and *occ*(*w*, *S*) = ∑_*v*∈*S *_*occ*(*w, v*). That is, mer_*k*_(*S*) is the set of different *k*-mers in all sequences from *S *and *occ*(*w, S*) is the number of positions in any sequence from *S *where *w *occurs.

### Occurrence ratios

For integers *q*, *q'*, 1 ≤ *q *≤ *q'*, the *k*-mer occurrence ratio of *S *is defined by

$$\rho_{S,k}(q,q^{\prime })=\frac{\left|\{w\in {\text{mer}}_{k}(S)|q\leq occ(w,S)\leq q^{\prime }\}\right|}{\left|{\text{mer}}_{k}(S)\right|}$$

That is, *ρ*_*S*, *k*_(*q*, *q'*) is the ratio of *k*-mers occurring between *q *and *q' *times in *S*. *ρ*_*S*, *k*_(1, 1) is the *k*-mer uniqueness ratio of *S*, i.e. the ratio of *k*-mers occurring exactly once in *S*. We define the multiple occurrence ratio by

$$\rho_{S,k}^{*}(q,q^{\prime })=\frac{\sum_{w\text{ is }k-\text{mer},q\leq occ(w,S)\leq q^{\prime }}occ(w,S)}{\sum_{w\text{ is }k-\text{mer}}occ(w,S)}.$$

Note that the denominator in this fraction equals the number of all positions in *S *where a *k*-mer occurs. The nominator restricts this count to positions where the corresponding *k*-mer occurs between *q *and *q' *times. While the occurrence ratio only considers the number of different *k*-mers, the multiple occurrence ratio takes the number of occurrences of a *k*-mer into account. We want to compute *ρ*_*S*, *k*_(*q*, *q'*) and $\rho_{S,k}^{*}$(*q*, *q'*) for a range of values of *k *and for any pair of values *q*, *q'*. We will later see how to do this efficiently.

### Average k-mer frequencies

The average frequency of *v *with respect to *S*, denoted by *λ*(*k*, *v*, *S*), is defined by

$$\lambda (k,v,S)=\log_{10}\frac{C(k,v,S)+1}{\left|{\text{mer}}_{k}(v)\right|}$$

where

$$C(k,v,S)=\sum_{w\in {\text{mer}}_{k}(v)}occ(w,S).$$

That is, *C*(*k*, *v*, *S*) is the sum of the frequencies of all *k*-mers in *v *with respect to *S*. Note that *λ*(*k*, *v*, *S*) is large if the *k*-mers in *v *occur many times in *S*. It is small if the *k*-mers in *v *rarely occur in *S*. Thus the average frequency measures how often the *k*-mers of some sequence *v *occur on average in some reference sequence set. If *v *is of length *k*, then it contains exactly one *k*-mer, namely *v*, which implies *λ*(*k*, *v*, *S*) = log_10_(*occ*(*v*, *S*) + 1).

### Distribution ratios

Now consider a set ℳS of sequence sets (we will later see that ℳS contains the first seven sets of Table 1). We want to compare the sequence data sets in ℳS according to the distribution of the average *k*-mer frequencies of the sequences they contain. The average *k*-mer frequencies are determined relative to some reference sequence set *S *(usually the 0.45 × WGS data set). To facilitate the comparison, we define non-overlapping subintervals of similar average *k*-mer frequency and calculate the fraction of sequences that belong to that subinterval (i.e. they have similar average *k*-mer frequency). As the sequence sets considerably differ in their size, we make these distributions comparable by computing ratios. To be precise, let

$$\lambda_{\min}=\min\{\lambda (k,v,S)|M\in ℳS,v\in M\}$$

and

$$\lambda_{\max}=\max\{\lambda (k,v,S)|M\in ℳS,v\in M\}$$

be the minimum and maximum possible *λ*-values for all sequences in the different sequence sets. We divide the interval [*λ*_min_, *λ*_max_] into equal sized non-overlapping subintervals at some suitable distance Δ. We obtain the intervals

[-*m*Δ, -(*m *- 1)Δ],[-(*m *- 1)Δ, -(*m *- 2)Δ],..., [(*n *- 2)Δ, (*n *- 1)Δ], [(*n *- 1)Δ, *n*Δ]

such that *λ*_min _= -*m*Δ and (*n *- 1)Δ ≤ *λ*_max _<*n*Δ. Then for each integer *j *∈ [-*m*, *n *- 1] we compute

$$\Omega_{k,M}(\lambda )=\frac{\left|\{v\in M|j\Delta \leq \lambda (k,v,S)<(j+1)\Delta \}\right|}{\left|M\right|}$$

where *λ *= *j*Δ. That is, Ω_*k*, *M *_is the fraction of sequences in *M *falling into the *j*th subinterval. Ω_*k*, *M *_is called *λ*-distribution ratio for *M*.

### Efficient computation of occurrence ratios

Note that the occurrence ratios only depend on the *k*-mers in *S*. That is, it is derived from the distribution of *occ*(*w, S*) for all *k*-mers *w *in *S*. Now let *δ*_*S*, *k *_be a table such that for all *i *≥ 1, *δ*_*S*, *k*_[*i*] is the number of different *k*-mers occurring exactly *i *times in *S*. For example, *δ*_*S*, *k*_[1] is the number of unique *k*-mers in *S*, i.e. the number of *k*-mers occurring exactly once in *S*. Then the following equations hold:

$$\begin{array}{ccc} \rho_{S,k}(q,q^{\prime }) & = & \frac{\sum_{i=q}^{q^{\prime }}\delta_{S,k}[i]}{\sum_{i>0,\delta_{S,k}[i]>0}\delta_{S,k}[i]}\\ \rho_{S,k}^{*}(q,q^{\prime }) & = & \frac{\sum_{i=q}^{q^{\prime }}i\cdot \delta_{S,k}[i]}{\sum_{i>0,\delta_{S,k}[i]>0}i\cdot \delta_{S,k}[i]} \end{array}$$

According to these equations we can efficiently compute *ρ*_*S*, *k*_(*q*, *q'*) and $\rho_{S,k}^{*}$(*q*, *q'*) from table *δ*_*S*, *k*_. To compute this, one needs to enumerate each *k*-mer with its occurrence count, thereby updating counters that were initialized to zero. That is, if a *k*-mer *w *with occurrence count *occ*(*w*, *S*) is enumerated, then one increments *δ*_*S*, *k*_[*occ*(*w*, *S*)] by one.

Traditionally, occurrence counts for *k*-mers are computed by hashing methods. However, these only work, if the number 4^*k *^of possible *k*-mers is considerably smaller than *n*. This does not hold for our application, where *k *ranges from 10 to 500. We have developed a different approach based on enhanced suffix arrays [21]. The space requirement and running time of our approach does not depend on the number of possible *k*-mers, but only on the total length of the sequences in *S*. Moreover, we can simultaneously compute *δ*_*S*, *k*_[*occ*(*w*, *S*)] for a range of values of *k*. This is useful when determining the optimal value for *k*.

To explain our approach we begin with the concept of enhanced suffix arrays, as introduced in [21].

#### Enhanced suffix arrays

Suppose *S *consists of *r *sequences. To process *S *we concatenate all sequences in *S *into a long string denoted $\bar{S}$ with unique separator symbols $_1_,...,$_*r*-1 _between the *r *sequences. Additionally we add a sentinel character $*r *following the last sequence in the concatenation. Obviously, $\bar{S}$ contains exactly the same *k*-mers as *S*. That is, we can compute *k*-mer counts based on $\bar{S}$.

Suppose that $\bar{S}$ is of length *n *+ 1. $\bar{S}$[*i*] denotes the character at position *i *in $\bar{S}$, for 0 ≤ *i *≤ *n *- 1. For *i *≤ *j*, $\bar{S}$[*i*..*j*] denotes the substring of *S *starting with the character at position *i *and ending with the character at position *j*. For *i *> *j*, $\bar{S}$[*i*..*j*] denotes the empty string. A substring of $\bar{S}$ beginning at the first position of $\bar{S}$ is a prefix of $\bar{S}$ and a substring ending at the last position of $\bar{S}$ is a suffix of $\bar{S}$. For each *h*, 0 ≤ *h *≤ *n*, $\bar{S}$_*h *_= $\bar{S}$[*h*..*n *- 1] denotes the *h*-th non-empty suffix of $\bar{S}$, i.e. the suffix beginning at position *h *in $\bar{S}$.

The key to our method is to lexicographically sort the suffixes of $\bar{S}$. Suppose that the characters are ordered such that A < C < G < T < N < $_1 _< ... <$_*r*_. This character order induces an order on all nonempty suffixes of $\bar{S}$, which is captured in the suffix array. The suffix array suf of $\bar{S}$ is an array of integers in the range 1 to *n*, specifying the lexicographic order of the *n *+ 1 non-empty suffixes of $\bar{S}$. In other words, ${\bar{S}}_{\text{suf}[0]},{\bar{S}}_{\text{suf}[1]},...{\bar{S}}_{\text{suf}[n]}$ is the sequence of suffixes of $\bar{S}$ in ascending lexicographic order.

The lcp-table lcp is an array of integers in the range 0 to *n*. For each *h*, 1 ≤ *h *≤ *n*, lcp[*h*] is the length of the longest common prefix of ${\bar{S}}_{\text{suf}[h-1]}$ and ${\bar{S}}_{\text{suf}[h]}$. Since = $_*r *_is the largest suffix in the lexicographic order, ${\bar{S}}_{\text{suf}[n]}$ = $_*r*_. Hence we always have lcp[*n*] = 0. In the following, the combination of the suffix array and the lcp-table is called enhanced suffix array.

The notion of lcp-intervals, introduced in [21] is central for the computation of the occurrence frequencies for the *k*-mers. An interval [*i*..*j*], 0 ≤ *i *<*j *≤ *n*, is an lcp-interval of lcp-value ℓ if

1. lcp[*i*] < ℓ,

2. lcp[*h*] ≥ ℓ for all *h*, *i *+ 1 ≤ *h *≤ *j*,

3. *h *= 0 or lcp[*h*] = ℓ for at least one *h *satisfying *i *+ 1 ≤ *h *≤ *j*,

4. *j *= *n *or lcp[*j *+ 1] < ℓ.

We will also use the shorthand ℓ-interval for an lcp-interval [*i*..*j*] of lcp-value ℓ. We say that an ℓ-interval [*i*..*j*] represents the substring $\bar{S}$[suf[*i*]..suf[*i*] + ℓ - 1] of $\bar{S}$ of length ℓ.

An interval [*i*..*i*], 0 ≤ *i *≤ *n *is a singleton interval. We say that a singleton interval [*i*..*i*] represents the suffix $\bar{S}$[suf[*i*]..*n *- 1] of $\bar{S}$. An *m*-interval [*l*..*r*] is said to be embedded in an ℓ-interval [*i*..*j*] if it is a subinterval of [*i*..*j*] (i.e., *i *≤ *l *<*r *≤ *j*) and *m *> ℓ. Note that we cannot have both *i *= *l *and *r *= *j *because *m *> ℓ. A singleton interval [*l*..*l*] is said to be embedded in an ℓ-interval [*i*..*j*], if *i *≤ *l *≤ *j*. If an interval [*l*..*r*] is embedded in [*i*..*j*], then [*i*..*j*] is the interval enclosing [*l*..*r*]. If [*i*..*j*] encloses [*l*..*r*] and there is no interval embedded in [*i*..*j*] that also encloses [*l*..*r*], then [*l*..*r*] is called a child interval of [*i*..*j*].

#### Enumerating k-mers and their occurrence counts

The parent-child relationship of the intervals constitutes a conceptual (or virtual) tree which we call the lcp-interval tree of the suffix array. The leaves of the tree are the singleton intervals and the internal nodes of the tree are the lcp-intervals. In particular, the root of this tree is the 0-interval [0..*n*]. An important property of the lcp-interval tree is the fact that it implicitly stores the number of occurrences of all substrings of $\bar{S}$. In particular, an interval [*i*..*j*] represents a string occurring *j *- *i *+ 1 times in $\bar{S}$. The idea is to read these occurrence counts from the lcp-interval tree. This works as follows: We use an algorithm described in [21] to enumerate the nodes of the lcp-interval tree. This algorithm has some important features:

(1) The nodes of the lcp-interval tree are enumerated in bottom-up order, i.e. a node, say *α*, is enumerated only after all nodes in the subtree below *α *have been enumerated.

(2) The children with the same parent node are enumerated according to the lexicographic order of the strings they represent.

(3) Whenever we process the children of a node, we have access to the lcp-value of the parent node.

(4) The values in tables suf and lcp are accessed in sequential order from left to right.

Due to property (2), the *k*-mers occurring in $\bar{S}$(represented by the intervals) are enumerated in lexicographic order. It now remains to show how to compute table *δ*_*S*, *k *_for a range of values *k *between user defined limits *k*_min _and *k*_max_.

Suppose that all values incremented below are initialized to zero.

• We process a singleton interval [*i*..*i*] as follows: Let *d *be the lcp-value of the parent node of [*i*..*i*]. Then $\bar{S}$[suf[*i*]..suf[*i*] + *k *- 1] is a *k*-mer occurring exactly once in $\bar{S}$ if and only if the following holds:

1. *d *<*k*,

2. suf[*i*] + *k *≤ *n*,

3. $\bar{S}$[suf[*i*]+*d*..suf[*i*] + *k *- 1] does not contain the symbol N.

As a consequence, for all *k*, max{*d *+ 1, *k*_min_} ≤ *k *≤ min{*k' *- 1, *k*_max_}, we increment *δ*_*S*, *k*_[1] by one, where *k' *is the minimum value greater than *d *such that either suf[*i*] + *k' *= *n *or $\bar{S}$[suf[*i*] + *k'*] = N.

• We process an ℓ-interval [*i*..*j*] different from [0..*n*] as follows: Let *d *be the lcp-value of the parent of [*i*..*j*]. Then $\bar{S}$[suf[*j*]..suf[*j*] + *k *- 1] is a *k*-mer occurring *j *- *i *+ 1 times in $\bar{S}$, if and only if *d *<*k *≤ ℓ. As a consequence, for all *k*, max{*d *+ 1, *k*_min_} ≤ *k *≤ min{ℓ, *k*_max_}, we increment *δ*_*S*, *k*_[*j *- *i *+ 1] by one.

#### Analysis of time and space requirement

The suffix array can be computed in linear time and space (cf. [30]). The same holds for the lcp-table, see [31].

The algorithm to enumerate the lcp-intervals and singleton intervals, given the enhanced suffix array, runs in linear time, see [21] for details. Whenever one visits a node, say *α*, one keeps track of all nodes on the path from the root to *α*. These are maintained on a stack, using constant time and space per node. In our specific application, we store the lcp-value and the left interval boundary of each node on the stack. Since one has access to the lcp-value of the parent node (see property (3)), one can process each lcp-interval in constant time. For the singleton intervals we need to verify that $\bar{S}$[suf[*i*] + *d*..suf[*i*] + *k *- 1] does not contain the symbol N. Rather than checking this condition character by character, we use information about ranges of Ns, preprocessed from the input sequence. That is, we store, in sorted order, the first position of each run of consecutive Ns in $\bar{S}$. Then for each position *i*, 0 ≤ *i *≤ *n *- 1, $\bar{S}$[*i*] ≠ N, we can determine the smallest position *i' *> *i *such that *i' *= *n *or $\bar{S}$[*i'*] = N using a binary search. Let *q *be the number of these runs. Then this method takes on the order of log_2_*q *time and requires extra space proportional to *q*.

Due to property (4), the enhanced suffix array does not need to be represented in main memory. At any time, we only need to store two consecutive entries of table suf and lcp. Hence the space requirement is dominated by the stack needed for the bottom-up traversal of the (virtual) lcp-interval tree. Our specific application only requires to store nodes representing strings of length shorter than *k*_max _occurring more than once as substrings in $\bar{S}$. Hence the stack size can be limited to *k*_max_, which results in a space requirement proportional to *k*_max_.

Besides random access to the sequence, we also need random access to a data structure for accumulating the occurrence counts. Let *τ *be the number of values *i *satisfying *δ*_*S*, *k*_[*i*] > 0 for some *k *∈ [*k*_min_, *k*_max_]. Then this data structure (e.g. hash table) requires space proportional to *τ *≤ *n*.

The overall space requirement of the algorithm is proportional to *n *+ *k*_max _+ *q *and the running time is proportional to *n *log_2_*q*. While the running time does not depend on *k*_max_, the space requirement is linearly dependent on *k*_max_. Since *k*_max _and *q *are both much smaller than *n*, they can be neglected. As a result, we obtain an algorithm that is linear in running time and space requirement. In contrast, the hashing methods run in time proportional to *n *+ 4^k ^time and space proportional to 4^k ^for some fixed value of *k*. That is, their running time and space requirement grows exponentially with *k*. As a consequence, these methods can only be applied for small values of *k*. These values are fixed, in contrast to our method which allows the computation for a range of values of *k*.

#### Dividing and merging the datasets

The analysis above shows that the running time and space requirement of our algorithm for computing *k*-mer counts (for ranges of values of *k*) is dominated by the suffix array construction. This is especially true for the space requirement. To get an idea of whether our method can be applied to large sequence sets, we have to consider the space requirement of the suffix array constructions in more detail. The most space efficient suffix array construction requires (*n*⌈log_2 _*n*⌉)/8 bytes per input symbol plus 2*n*/8 bytes for representing the sequence. Given a 32-bit computer with 4 gigabytes (2^32 ^- 1 bytes) of main memory, *n *has to satisfy the inequality (2*n *+ *n*⌈log_2 _*n*⌉)/8 ≤ 2^32 ^- 1. That is, the sequence length is limited to 1 gigabyte. Since we want to process considerably larger sequences, we developed a divide-and-conquer approach. This cuts the sequence $\bar{S}$ into sufficiently small non-overlapping sections, such that for each section we can compute the corresponding enhanced suffix array on a 32-bit computer (equipped with 4 gigabytes of main memory).

Processing each section by the algorithm described above results in occurrence counts for each section. More precisely, for each section we enumerate pairs (*occ, pos*) where *pos *is a position in the corresponding section and *occ *is the number of occurrences of $\bar{S}$ [*pos*..*pos *+ *k *- 1] in this section. Thus each pair encodes a *k*-mer, and the pairs are stored on a file in the order they are delivered by the algorithm, namely in lexicographic order with respect to the *k*-mer. This property allows to combine the appropriate values in a merging process, which works as follows: Suppose we are given, say *m*, files storing the occurrence counts for the *m *sections of $\bar{S}$. These files are merged in *m *- 1 pairwise merge-operations. The merging steps only require to have the entire sequence $\bar{S}$ represented in main memory. Since we restrict to DNA sequences and, by construction, do not process any substrings of $\bar{S}$ containing the character N, we can store each nucleotide in 2 bits, i.e. *n*/4 bytes suffices for representing the sequence. That is, the merging procedure allows to process sequences of length up to 16 gigabytes on a 32-bit computer.

### Efficient computation of distribution ratios

In contrast to the occurrence ratios, the average frequency *λ*(*k*, *v*, *S*) of a sequence *v *is determined relative to the *k*-mer content of *S*. Since we want to compute *λ*(*k*, *v*, *S*) for a fixed set *S *and many different sequences *v*, it makes sense to preprocess *S *into a *k*-mer frequency index I(*S*, *occ*_min_, *occ*_max_) storing all *k*-mers occurring between *occ*_min _and *occ*_max _times in *S*. Here *occ*_min _and *occ*_max _are user defined positive numbers. Constraining the indexed *k*-mers by user specified values *occ*_min _and *occ*_max _is relevant in applications where we are interested in *k*-mers occurring rarely (small value of *occ*_max_) or frequently (large value of *occ*_min_). Given the index I(*S*, *occ*_min_, *occ*_max_), we want to solve the following tasks:

(1) For each possible sequence *w *of length *k*, determine if it occurs in the index.

(2) For each *k*-mer occurring in the index, determine the number of its occurrences in the index.

In the previous section we have shown how to compute occurrence ratios by enumerating *k*-mers. Instead of deriving occurrence ratios by incrementing some table *δ*_*S*, *k*_, the same enumeration process can determine the *k*-mers *w *satisfying the constraint *occ*_min _≤ *occ*(*w, S*) ≤ *occ*_max_. If this is satisfied, the *k*-mer is stored on a file. As the *k*-mers are enumerated in lexicographic order, the index I(*S*, *occ*_min_, *occ*_max_) is simply a sequence of lexico-graphically ordered *k*-mers stored on a file. Whenever this is needed it can be mapped into main memory.

**Figure 1:**
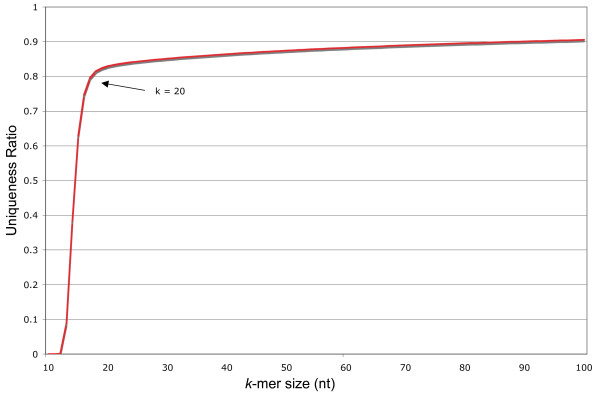
***K*****-mer uniqueness ratio for the 0.45 × maize WGS data set for varying values of*****k***. The uniqueness ratio is the ratio of *k*-mers occurring exactly once relative to all *k*-mers in the set. *k *= 20 balances the information content with *k*-mer resolution, visible as a natural inflection point on the curve which may change with organism, sequencing technology, and coverage employed.

To implement I(*S*, *occ*_min_, *occ*_max_), we directly store each *k*-mer together with the occurrence count, if this is required. As a *k*-mer is a string over an alphabet of size 4, it can be stored in *k *log_2 _4 = 2*k *bits. This, of course restricts the choice of *k *to small values. But since the optimal values for *k *are rather small (see Figure 1), this is not a restriction in practice.

Let *r *be the number of indexed *k*-mers. Since the stored *k*-mers are lexicographically ordered, for each possible *k*-mer, task (1) and (2) can be solved by a binary search over the index. Each step of the binary search requires to compare 2*k *bits. As these are represented by integers of the machine word size *ω*, a single comparison takes time proportional to $\left\lceil \frac{2k}{\omega }\right\rceil$. There are at most log_2 _*r *comparisons to determine if a *k*-mer occurs in the index. If it occurs, then the occurrence count can be determined in constant time by a single table lookup. Altogether, the search for a *k*-mer takes time proportional to $\left\lceil \frac{2k}{\omega }\right\rceil$ log_2 _*r*. For example, on a 32-bit computer, it takes on the order of 2 log_2 _*r *steps to perform the binary search when *k *= 20.

To put it together, our index differs in several aspects from other indexing approaches employed in sequence analysis (e.g. suffix trees [8], suffix arrays [32], or hash tables):

• First, we do not store information about where the *k*-mers occur in *S*. This fact leads to some simplifications for the implementation of the index and allows faster querying time. Moreover, it means that the size of the index is not dependent on the size of *S*. It rather depends on the choice of values *occ*_min _and *occ*_max_. For large sequence sets and larger values of *k*, the number of different *k*-mers becomes very large. When computing occurrence ratios, this is not a problem, as only *k*-mer counts are accumulated. However, when constructing the *k*-mer index, *occ*_min _and *occ*_max _should be chosen carefully. If these values are not restrictive enough, then there may be too many *k*-mers to be indexed. That is, the input sequence representation and the *k*-mer index may not fit into memory any more.

• Second, we directly store each *k*-mer as a bit string together with the occurrence count in the index. That is, the information about the indexed *k*-mer is not distributed over different memory locations, as in other indexing approaches (which usually store pointers into the original sequence). As a consequence, the querying times for our index are extremely short.

The Tallymer software [33] provides programs for computing occurrence ratios (*tallymer-occratio*), for generating a *k*-mer index (*tallymer-mkindex*), and for searching a *k*-mer index (*tallymer-search*). The software-distribution is complemented by a program to construct enhanced suffix arrays. The 32-bit version of this program can handle sequences of size about 150 million bp per gigabytes of main memory. For example, to handle the 0.45 × WGS data set (total length 1,088,525,270 bp.) in this amount of main memory, we have to split it into eight sections each of approximately 136 million bp. The program *tallymer-occratio *provides flexible options to specify the range of *k*-mer sizes considered and to tailor the values output. For example, one can specify that values are output as ratios instead of absolute counts. Comparison of *k*-mer frequencies of two sequence sets is not directly supported by the software, but can easily be done as follows: create an index of the first sequence set and query it with the second, and vice versa.

The program *tallymer-search *takes multiple fasta files as input. It generates all *k*-mers not containing any N from the given files, and matches them against the index in forward and/or reverse direction. Currently only exact matches are supported. An extension to degenerate matches would require a combination of index traversal and dynamic programming techniques, as, for example, implemented in Vmatch [9] for the case that the index is an enhanced suffix array.

## Results and discussion

### Selection of k-mer size for use in maize

Because our method allows us to compute *k*-mer frequencies for large values of *k*, we have a wide latitude for selection of a *k*-mer size. Figure 1 plots the *k*-mer uniqueness ratio for the 0.45 × WGS for *k *in the range 10 to 500. As *k *approaches 500, the curve reaches unity. The information content of the *k*-mer set increases at a very fast rate from *k *= 10 to *k *= 20. Beyond this point, increasing *k *does not significantly increase the number of unique *k*-mers, but does decrease the overall resolution of the *k*-mer set. The inflection point on this curve is likely to change for other genomes and other sources of survey sequence, but for our WGS reference, *k *= 20 is optimal.

### Validation of the 20-mer frequency index for the WGS set

The use of *k*-mer frequencies is premised on the availability of an unbiased sequence set reflecting the overall repeat character of the genome in question. To test whether the 0.45 × WGS meets this criterion, we computed the corresponding 20-mer frequency index, i.e. *S *is the 0.45 × WGS set and *k *= 20. It contains 456,445,768 different 20-mers. There are 1,041,350,089 positions at which a 20-mer occurs. We screened seven publicly available maize sequence sets (see Table 1), against *S*. That is, we evaluated Ω_*k*, *M *_for *M *∈ {BAC, BES, RepI, RepII, EST, AZM4 HC, AZM4 MF}.

The public maize sequence sets fall into three classes: (A) maize whole genome sequences, (B) maize repeats, and (c) maize gene enrichment sequences. The seven sequence sets are known to have differing degrees of repetitiveness and should therefore provide a means to verify our method. For example, we expect gene enriched sequence to be less repetitive than RepII sequences (DNA transposons), and RepII sequences to be less repetitive than RepI sequences (TEs).

**Figure 2:**
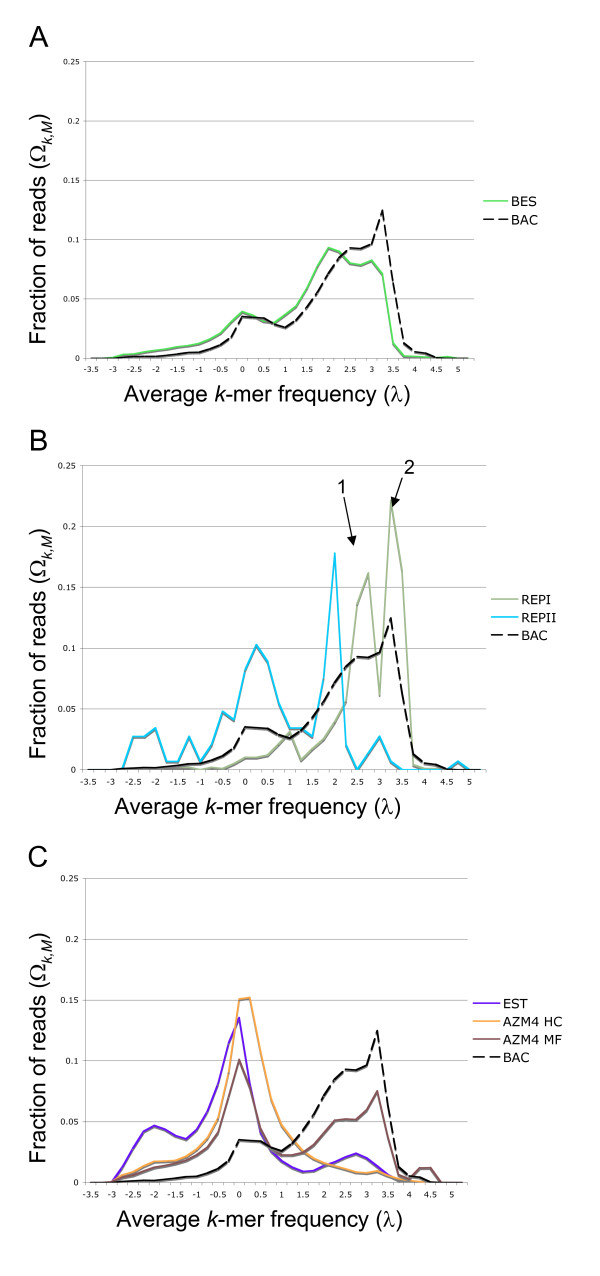
***λ*****-distribution ratios for different classes of maize sequences. **The X-axis shows the *λ* -values between *λ*_min _and *λ*_min_. The Y-axis shows the values for Ω_*k*, *M*_, where *M *is one of the first seven sequence sets from Table 1. Three sequence classes are shown: (A) Whole Genome Sequences, (B) Repeat Sequences, and (C) Gene Enrichment Sequences. The BAC profile is provided as a reference in all three panels. In (B) the two peaks in the bimodal distribution of the RepI curve are marked by the numbers 1 and 2, see also Table 2.

The results of this analysis are shown in Figure 2. Panel A confirms that the two maize whole genome sequence sets have similar frequency distributions, providing an overall, unbiased repeat profile for the maize genome. The λ-distribution ratios of RepI, RepII, and BAC sequence is shown in Panel B. RepI repeats exhibit high average frequency, many of which exceed 100 copies in the 0.45 × WGS set, while the RepII repeats, enriched in low-copy elements, are far less repetitive. Notably, the λ-distribution ratios for both repeat classes are bimodal. The more repetitive peak for RepI corresponds to an enrichment of Ty3/gypsy repeat elements, while the less repetitive reflects enrichment in Ty1/copia (see Table 2, ($p_{\chi^{2}}$ = 1.9·10^-57^)). Though the λ-distribution for RepII also appears to be bimodal, we were unable to find significantly different repeat populations among the DNA transposon derivatives and superfamilies.

**Table 2:** Two repeat populations in the RepI sequence set. Repeat elements of the RepI set are found in different relative proportions depending on the repeat level tested (corresponding to peaks 1 and 2 in Figure 2B).

	peak 1		peak 2	
class	total	percentage	total	percentage
unclassified	66	11.0	1	0.3

LINE	8	1.3	0	0

Ty1/copia	142	23.6	292	75.5

Ty3/gypsy	386	64.1	94	24.3

	∑ 602	∑ 100.0	∑ 387	∑ 100.0

The three sequence sets delivered by gene enrichment methods shown in Figure 2C peak around an average frequency of 0 in the WGS set. The relative uniqueness of these reads justifies the attribute 'gene-enriched', but their apparent efficacy varies significantly. The High-*C*_0_*t *method succeeds in concentrating unique sequence without much leakage of the highly repetitive content evident to some extent in the ESTs, and to a far greater extent in the methyl-filtered library. This difference observed between the High-*C*_0_*t *and methyl-filtered libraries was previously observed in [34].

### Genome annotation using k-mer frequencies

**Figure 3:**
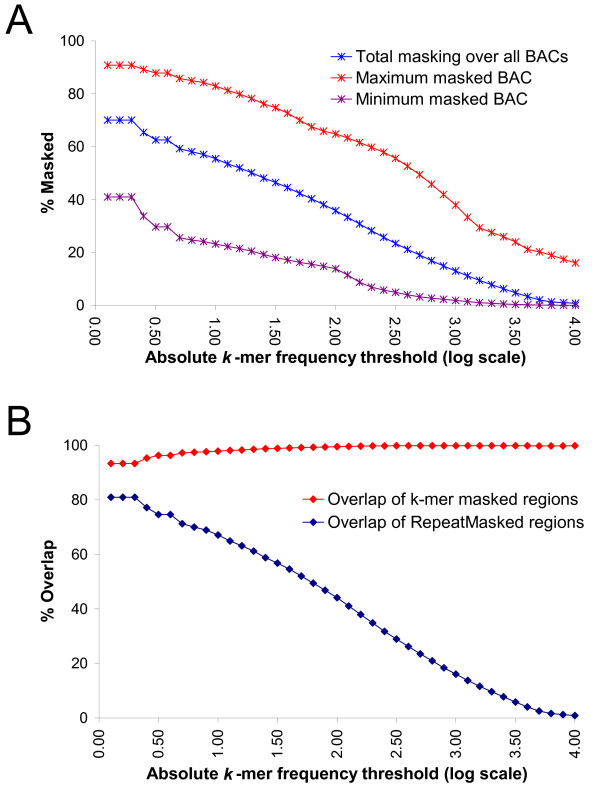
**Comparison of masking using either *k*-mer frequencies or alignment-based repeat masking.** (A) Percent of nucleotides masked in 100 BAC sequences (total length 14.3 Mb) as a function of absolute frequency threshold (logarithmic scale). Values are given for the sum of all sequences, and for the most and least repetitive BACs within the set. (B) Overlap between regions masked using the *k*-mer frequency based method and those masked using RepeatMasker (MIPS REcat library).

We used a previously published set of 100 maize BAC sequences that had been chosen at random to be representative of the whole genome [35]. A position, say *i*, in a BAC was masked if the logarithm of the absolute frequency of the 20-mer starting at position *i *achieves some threshold. Figure 3A shows that, summing over all BACs, 50% of nucleotides were masked at an absolute frequency threshold of 1.3 or greater, corresponding to at least 20 occurrences in the WGS index. Coverage of individual BACs at this threshold ranged from 20.5% to 78.3%. Reducing the threshold resulted in greater coverage. At the lowest threshold of 0.3 (i.e. 2 or more copies), total coverage reached 70.1% (range for individual clones, 41.0–90.8%).

We compared these results to masking based on the curated MIPS REcat repeat library. This library includes repeats from many sources including annotated TEs from the BACs used in this analysis. Thus application of this library can be regarded as a 'gold-standard' for detection of TEs within these BACs. Indeed, masking using the MIPS REcat library, resulted in a total repeat coverage of 80.8% (range 36.8–100.0% on individual clones). This exceeds the masking rate of 67% originally reported for this set [35], suggesting that the MIPS REcat library has been updated with new repeat sequences after its publication in 2005. Figure 3B shows the extent of overlap between masked positions using these two methods. Overlap of positions masked by our method with curated TEs in REcat exceeded 93% at lowest absolute frequency threshold and reached a maximum of 99.85% over the most repetitive regions (threshold ≥ 4.00). Overlap was 98.51% at an absolute frequency threshold of 1.3. These results indicate that the MIPS REcat library is on the whole inclusive of moderately and highly repetitive regions in these BACs.

In contrast, 81% of the MIPS REcat-masked positions are also masked by our method. This was achieved at the absolute frequency threshold of 0.3 (Figure 3B). At the absolute frequency threshold of 1.3, only 61.2% of positions masked by MIPS REcat were also masked by our method.

We next compared the two repeat detection methods for their ability to discriminate TEs at the level of predicted genes. The set of 100 BACs were annotated using FGENESH and resulting predictions were classified as presumptive genes or as TEs using a similarity-based search (see Section "Methods"). Of the 2504 predicted genes, 359 (14%) were screened out as showing no evidence of homology to NCBI GenPept peptides. Of the remainder, 1842 (86%) were classified as TE while the remaining 303 (14%) were classified as presumptive genes. For these latter two classes the percent of coding sequence masked was calculated based on either RepeatMasker data or on constituent 20-mer frequencies at various thresholds. For each method of masking, receiver operating characteristic (ROC) curves were used to define a threshold of masking that best discriminated TEs from presumptive genes. Area under the curve (AUC), sensitivity, and specificity were used to compare efficacies [29].

**Figure 4:**
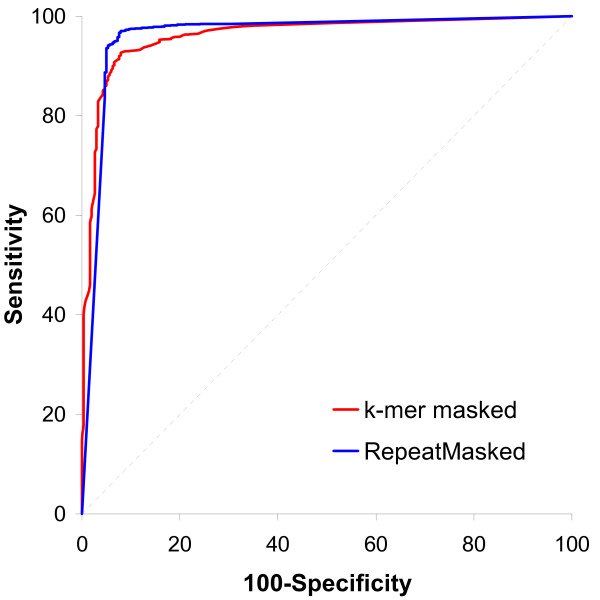
**ROC plots showing sensitivity and specificity of TE detection among 2145 FGENESH models (1824 TE and 303 presumed genes) based on the percent of coding sequence masked using two methods.** In one method BAC sequences were masked using an absolute frequency threshold of 0.8. In the other, masking was performed using RepeatMasker with the MIPS REcat library. ROC plot comparison of the maximum area under the curve resulting from the two plots showed that they are not significantly different (see main text for details).

Because percent masking using *k*-mers depends on which absolute frequency threshold is used, we first optimized the threshold by comparing all ROC curves for absolute frequency thresholds between 0 and 4.0 at 0.1 increments. The threshold of 0.8 gave the maximum area under the curve (AUC), with a value of 0.961 (95% CI 0.952 to 0.969, p-value = 0.0001). For masking using REcat the AUC was only slightly higher, 0.962 (95% CI 0.953 to 0.969, p-value = 0.0001). Comparison of these two ROC curves, shown in Figure 4, revealed that this difference in AUC was not significant (p-value = 0.967).

Table 3 compares the sensitivity and specificity of the two methods for detecting TE-encoded genes. With a sensitivity of greater than 96%, REcat masking was better able to detect TE genes than our method. This was expected since the TEs within these BAC sequences had been previously annotated and are constituents of the REcat database. Nevertheless our method was able to detect greater than 92% of TE genes. This demonstrates that a substantial proportion of TE genes, approaching that based on annotation, can be detected using *k*-mer frequencies as found within a low coverage whole genome shotgun sequence. Furthermore, the two methods showed a similar false-positive rate, each having specificity near 92%.

**Table 3:** Discrimination of maize TE-encoded genes based on percent of coding sequence masked using either RepeatMasker (MIPS REcat library) or constituent 20-mer frequencies (WGS index with a threshold log repeat level of 0.8).

method	criterion	sensitivity	95% CI	specificity	95% CI
REcat	> 41.56	96.69	95.8–97.5	92.41	88.8–95.1
*k*-mer frequency	> 17.00	92.62	91.3–93.8	92.08	88.4–94.9

*Ab initio *gene prediction was carried out on 100 non-masked BAC sequences using FGENESH, and resulting models were classified as TE (1842) or as presumed genes (303) based on a BLASTP similarity search. ROC analysis was used to determine the optimum criterion (threshold percent of coding sequence masked) that would maximize detection of TEs while minimizing false positives (i.e. maximum sensitivity + specificity).

While both RepeatMasker and our method masked the majority of RepI retroelements, some low copy TEs escaped masking under our method based on average frequencies. As shown in our analysis of the 100 pilot BACs, low-copy DNA transposons, may be annotated as such by curated repeat databases, but missed by the counting approach used here. In the context of directed sequence finishing, low-copy repeats are often as in need of characterization as protein coding genes. Leaving them unmasked in maize is actually in the best interests of the project. But the average frequency threshold must be chosen carefully: more permissive thresholds will lead to finishing TE-like elements, and more strict thresholds may mask high-copy gene families. To use this method optimally, a balance must be struck with respect to the genome in question, and the thresholds need to be adjusted according to the annotation requirements.

**Figure 5:**
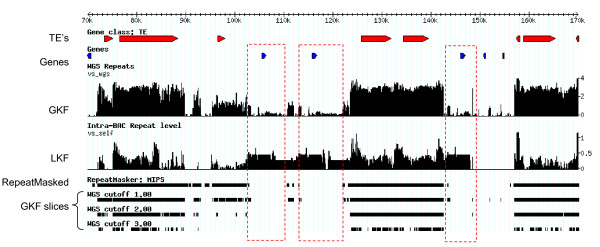
**Visualization of *k*-mer frequencies in a 453 kbp assembly of four BAC sequences derived from maize chromosome 8. **A 100 kbp segment (range 70,001–170,000 nt) is shown. In the first two tracks transposable elements are shown in red while genes are shown in blue (exon/intron structure not shown). The third track, global *k*-mer frequency (GKF), shows for each position of the mentioned region (X-axis) the average frequencies *λ*(*k*, *v*, *S*) (Y-axis) of the *k*-mer *v *beginning at this position. Here *S *is the 0.45 × WGS set mentioned above. The fourth track, local *k*-mer frequency (LKF), shows *λ*(*k*, *v*, *R*), where *R *is the larger 453 kbp region under scrutiny. RepeatMasker results using the MIPS REcat repeat libraries are given alongside sequence masked using absolute frequency thresholds of 1, 2, and 3. Three genes (boxed) related to a selenium binding protein apparently arose by tandem duplication and have high LKF compared to other non-TE genes in the assembly.

The validated WGS index can be used to annotate any portion of the genome with respect to its component *k*-mer frequencies. In another set of experiments, we analyzed a 453 kb portion of maize chromosome 8 (assembled in-house as described in Section "Sequence data sets") and display a portion of its annotation from position 67,000 to 165,000 in Figure 5. The first two tracks show the locations of FGENESH *ab initio *gene predictions classified as either putative protein-coding genes (blue) or transposable elements (red). The Global *k*-mer Frequency (GKF) track visualizes the absolute frequency across the region. Frequencies in this histogram were generated by querying each overlapping 20-mer in a 5' to 3' direction against the WGS index. Note that some regions contain *k*-mers occurring on the order of 1000 times in the maize WGS set. In the GKF track, TE-like genes often correspond to regions of high absolute frequency, while genes similar to known proteins reside in regions of smaller absolute frequencies.

If the scope of the experiment is narrowed, however, and the *k*-mer frequencies are computed with respect to the region itself, independent of the WGS index, a significantly different picture emerges. The Local *k*-mer Frequency (LKF) track represents a self-analysis: the frequencies of overlapping 20-mers in the query are determined relative to the 453 kb region alone. Even in a local context many TEs are present at high copy number (the LKF track also shows values on a logarithmic scale). But the LKF highlights local features in a way GKF cannot. This region contains four locally duplicated genes related to Selenium-binding proteins. Figure 5 shows that these genes have a higher LKF than surrounding genes that are present at single copy. Such peaks are not evident in the GKF track indicating that these genes have low overall copy number within the genome. This analysis could be employed to identify local expansions of paralogous genes commonly found in plant genomes [36].

### Comparative genomics

**Figure 6:**
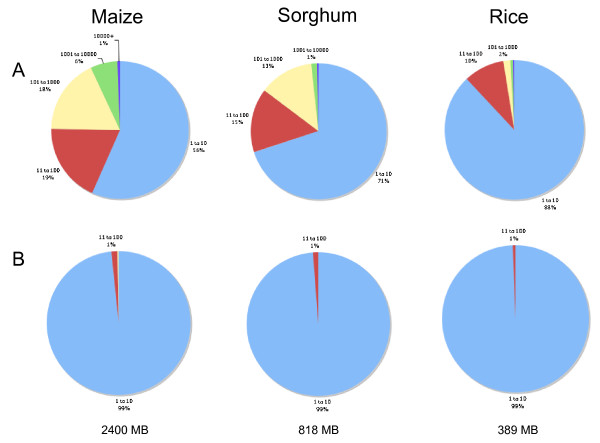
**Occurrence ratios in comparative genomics.** Maize, sorghum and rice whole genome shotgun reads were randomly selected to generate 0.45 × coverage with respect to each genome's size. The total number of 20-mers in each logarithmic frequency class (A) are contrasted to the number of different 20-mers in each frequency class (B). Maize is the most repetitive of the three grasses analyzed here, but a corresponding increase in genome complexity is not observed.

Beyond employing *k*-mer frequencies to annotate sequence with copy-number information, we have found that the frequency information contained therein are themselves biologically informative, illuminating cross species differences in repetitive content. For example, Figure 6 compares whole genome shotgun sets acquired from three distinct sequencing projects: rice [37], sorghum [38], and the 0.45 × maize set (JGI) employed throughout this analysis. Using randomly selected reads to simulate 0.45 × coverage in each species given their predicted genome sizes [39-41], we computed (multiple) occurrence ratios, i.e. percentage values 100·$\rho_{S,k}^{*}$(1, 10) and 100·$\rho_{S,k}^{*}$(11, 100) (Figure 6A) and 100·*ρ*_*S*, *k*_(1, 10), 100·*ρ*_*S*, *k*_(11, 100), 100·*ρ*_*S*, *k*_(101, 1000), 100·*ρ*_*S*, *k*_(1001, 10000), 100·*ρ*_*S*, *k*_(10001, ∞) (Figure 6B), where *k *= 20 and *S *is the respective sequence set. Recall that the occurrence ratio *ρ*_*S*, *k*_(*q*, *q'*) is the ratio of *k*-mers occurring between *q *and *q' *times in *S*. The multiple occurrence ratio $\rho_{S,k}^{*}$(*q*, *q'*) is similarly defined, but takes the number of occurrences of a *k*-mer into account. See section "Methods" for details.

For example, in the case of maize, there are 1,041,350,089 positions at which a 20 mer occurs. There are 456,445,768 different 20-mers of which 378,556,535 are found only once, while the most highly represented sequences exists 47,933 times.

The multiple occurrence ratios represented in Figure 6A show that maize contains the most repetitive sequence, followed by sorghum, and rice. Nearly 25% of maize 20-mers occur more than 100 times while only 14% of sorghum and 13% of rice 20-mers exhibit this frequency. This finding was expected. When only considering the number of different 20-mers in Figure 6B, we find that a mere 1% of all 20-mers occurs more than 10 times, i.e. accounts for the repetitive fractions. This is a remarkable equivalence in complexity across the three organisms. Low complexity is consistent with relatively recent proliferation of a small number of originating TE's resulting in limited divergence of *k*-mers. Maize demonstrates the most substantial growth in repetitive content, a finding attributable to its known TE expansion [3-5]. Thus, the *k*-mer frequency method provides a novel way of quantifying this important evolutionary event.

**Figure 7:**
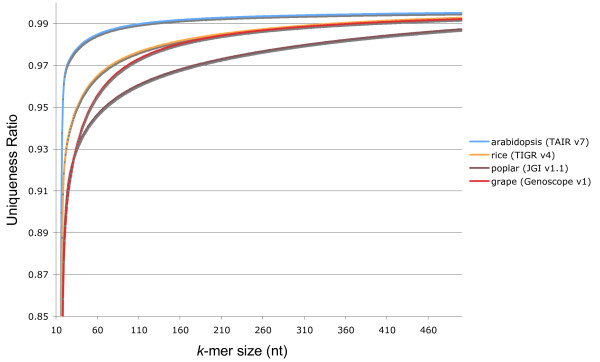
**The *k*-mer uniqueness ratio for some assembled plant genomes as a function of *k*.** The uniqueness ratio is the ratio of *k*-mers occurring exactly once relative to all *k*-mers in the set. It is computed for every *k *between 10 and 500. Extrapolating beyond the tested *k*-mer interval, it appears as though poplar, rice, and grape approach unity at a much slower rate than arabidopsis.

Read lengths in whole genome shotgun sequencing projects limit this sort of analysis. Since Sanger reads average around 700 base pairs in length, most repetitive elements will be truncated at the 5' and 3' ends, making experiments with *k*-mer sizes greater than 50 base pairs impractical. However, genome assemblies obviously do not suffer from these read length restrictions. Currently, there are very few plant assemblies. Though a number of projects are underway, only four genomes have been published: arabidopsis [42], rice [37,40], poplar [43], and grapevine [44]. In Figure 7 we plot the *k*-mer uniqueness ratio for these genomes, as a function of *k*. We tested *k*-mer sizes from *k *= 10 to *k *= 500. With *k *approaching 500, the uniqueness ratio converges to 1. More repetitive genomes such as rice and poplar converge at a much slower rate than arabidopsis.

**Figure 8:**
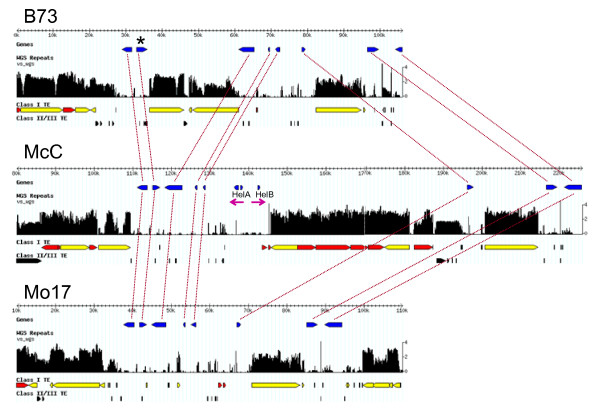
***K*****-mer frequencies across orthologous regions of three maize cultivars.** The B73-based WGS index was used to annotate the Bronze-1 locus and surrounding regions in cultivars B73, McC and Mo17 (Genbank accession numbers AF448416, AF391808, and AY664416, respectively). Orthologous genes present in all three cultivars are connected with red lines. The Bronze-1 locus is shown with an asterisk. Helitrons HelA and HelB in McC, were previously described by [45]. Ty1/copia retrotransposons are shown in red while those of the Ty3/gypsy class are shown in yellow, as classified using MIPS REcat masking. Though the transposition histories vary across the three cultivars, the frequency index can successfully be used to annotate the repeat regions in McC and Mo17.

We performed a number of experiments demonstrating that *k*-mer frequencies cannot be used to annotate repeats across species (data not shown) owing to divergent TE families. But within a species, our method seems to work across cultivars regardless of which subtype is considered. For maize, our standard 0.45 × WGS sequence set is derived from B73, the cultivar currently being sequenced. Figure 8 shows that using this index, one can successfully mask the TEs of two sister cultivars, McC and Mo17, in the well-characterized Bronze-1 locus [45]. While the transposition histories of the three cultivars differ markedly, the *k*-mers in these TEs have undergone little divergence.

## Conclusion

We have described a method based on *k*-mer frequencies allowing for annotating large repetitive plant genomes. As we have demonstrated, our method is useful as an alternative or supplementary form of repeat annotation in novel genomes. The novelty of genomes being sequenced is precisely the reason why this approach has value. By definition, new genomes lack comprehensive repeat libraries, and the construction of de novo libraries is often hampered by the paucity of sequence available at a project's outset. In the absence of such libraries, survey sequences in the form of Sanger WGS, 454 [46], Solexa [47], or SOLID [48] reads can be used to create cheap, abbreviated *k*-mer frequency indices for use across multiple subtypes.

In designing the *k*-mer frequency approach to be completely independent of any manual annotation, we apply a binary decision framework: given a numerical threshold, sequence is designated as either repeat or non-repeat. Though binary designations are sufficient for sequence masking, we believe that the *k*-mer frequencies (and measures we derive from it) are best used in combination with other methods designed for careful repeat annotation as demonstrated in the accompanying publication [49].

To apply our methods to large sequence sets, we have developed fast and memory efficient algorithms to compute occurrence ratios, to index *k*-mers, and to retrieve their occurrence counts from the index. The algorithms are implemented in the Tallymer software. A distribution of the software and the Perl-scripts post processing the output is available from the Tallymer website [33].

## Authors' contributions

SK developed the algorithms and implemented the Tallymer software. AN analyzed sequence data sets with respect to Tallymer output. JS performed annotation of maize BACs, their visualization, and ROC analysis. DW conceived this study. All authors contributed to writing the article.
